# Supplementary material for: Distinct lateral inhibitory circuits drive parallel processing of sensory information in the mammalian olfactory bulb
Source: eLife. 2016 Jun 28;5:e16039. doi: 10.7554/eLife.16039 (PMC4972542; doi:10.7554/eLife.16039)
Supplement: Figure 4—source data 1. — DOI: http://dx.doi.org/10.7554/eLife.16039.012 [file elife-16039-fig4-data1.docx]

**Figure 4 – source data 1: Tables of GC properties**

| **Supplementary Table 1. Morphological properties of GCs** | | | |
| --- | --- | --- | --- |
|  | sGC | dGC | *P* value |
| Soma area (μm^2^) | 74.9 ± 25.9 (19) | 78.6 ± 17.3 (11) | 0.67 (n.s.) |
| Soma depth from MCL (μm) | 24.9 ± 26.6 (19) | 75.5 ± 21.6 (11) | 1.0×10^-5^ *** |
| Basal dendrites, Σ length (μm) | 217.8 ± 186.0 (19) | 177.6 ± 121.9 (11) | 0.53 (n.s.) |
| Basal dendrites, Σ volume (μm^3^) | 70.1 ± 62.3 (19) | 65.2 ± 76.0 (11) | 0.85 (n.s.) |
| Apical dendrites, Σ length (μm) | 1890.6 ± 477.1 (19) | 1642.5 ± 587.0 (11) | 0.22 (n.s.) |
| Apical dendrites, Σ volume (μm^3^) | 642.5 ± 282.3 (19) | 606.7 ± 222.7 (11) | 0.72 (n.s.) |
| Apical dendrites, gemmules | 241.9 ± 81.0 (19) | 203.1 ± 138.7 (11) | 0.34 (n.s.) |
| Values reported are mean ± standard deviation (*n*). *p<0.05; **p<0.01; ***p<0.001; n.s., not significant (two-tailed unpaired *t* test). | | | |

| **Supplementary Table 2. Action potential properties of GCs** | | | |
| --- | --- | --- | --- |
|  | sGC | dGC | *P* value |
| V_threshold_ (mV) | –31.9 ± 6.3 (19) | –23.8 ± 5.7 (8) | 4.8×10^-3^ ** |
| Amplitude (mV) | 60.6 ± 8.8 (19) | 44.3 ± 7.6 (8) | 1.1×10^-4^ *** |
| FWHM (ms) | 0.97 ± 0.14 (19) | 1.09 ± 0.29 (8) | 0.16 (n.s.) |
| Rising slope (mV ms^-1^) | 195.9 ± 53.5 (19) | 109.4 ± 33.9 (8) | 2.9×10^-4^ *** |
| Falling slope (mV ms^-1^) | –63.2 ± 12.8 (19) | –53.8 ± 17.2 (8) | 0.13 (n.s.) |
| Values reported are mean ± standard deviation (*n*). *p<0.05; **p<0.01; ***p<0.001; n.s., not significant (two-tailed unpaired *t* test). | | | |

| **Supplementary Table 3. Spike train properties of GCs** | | | |
| --- | --- | --- | --- |
|  | sGC | dGC | *P* value |
| Rheobase (pA) | 36.3 ± 20.9 (19) | 46.2 ± 22.6 (8) | 0.28 (n.s.) |
| Rheobase first-spike latency (ms) | 543.6 ± 542.9 (19) | 209.0 ± 164.9 (8) | 0.10 (n.s.) |
| Gain (Hz pA^-1^) | 0.86 ± 0.33 (19) | 0.91 ± 0.34 (7) | 0.74 (n.s.) |
| Peak instantaneous rate (Hz) | 59.3 ± 23.5 (19) | 42.4 ± 15.2 (8) | 0.074 (n.s.) |
| Values reported are mean ± standard deviation (*n*). *p<0.05; **p<0.01; ***p<0.001; n.s., not significant (two-tailed unpaired *t* test). | | | |

| **Supplementary Table 4. Passive membrane properties of GCs** | | | | | | |  |
| --- | --- | --- | --- | --- | --- | --- | --- |
|  | sGC | | dGC | | *P* value | |  |
| R_input_ (MΩ) | 599.8 ± 397.6 (20) | | 499.7 ± 248.9 (8) | | 0.52 (n.s.) | |  |
| τ_m_ (ms) | 26.6 ± 14.6 (17) | | 26.1 ± 11.5 (7) | | 0.94 (n.s.) | |  |
| C_m_ (pF) | 46.0 ± 12.2 (17) | | 48.9 ± 11.5 (7) | | 0.60 (n.s.) | |  |
| V_rest_ (mV) | –71.3 ± 6.8 (22) | | –65.6 ± 10.0 (8) | | 0.085 (n.s.) | |  |
| Values reported are mean ± standard deviation (*n*). *p<0.05; **p<0.01; ***p<0.001; n.s., not significant (two-tailed unpaired *t* test). | | | | | | |  |
| **Supplementary Table 5. Spontaneous synaptic event properties of GCs** | | | | | | | |
|  | | sGC | | dGC | | *P* value | |
| sEPSP | |  | |  | |  | |
| *Frequency (Hz)* | | 7.4 ± 3.0 (21) | | 6.6 ± 2.0 (8) | | 0.45 (n.s.) | |
| *Amplitude (pA)* | | 1.2 ± 0.5 (21) | | 0.9 ± 0.3 (8) | | 0.09 (n.s.) | |
| *Rise_20-80%_ (ms)* | | 2.7 ± 0.6 (21) | | 2.6 ± 0.3 (8) | | 0.86 (n.s.) | |
| *τ_decay_ (ms)* | | 19.4 ± 3.9 (21) | | 26.4 ± 15.8 (8) | | 0.066 (n.s.) | |
| sEPSC | |  | |  | |  | |
| *Frequency (Hz)* | | 4.2 ± 4.2 (26) | | 4.0 ± 4.8 (10) | | 0.87 (n.s.) | |
| *Amplitude (pA)* | | –18.0 ± 7.1 (26) | | –14.8 ± 7.1 (10) | | 0.24 (n.s.) | |
| *Rise_10-90%_ (ms)* | | 1.0 ± 0.2 (26) | | 1.2 ± 0.4 (10) | | 0.035 * | |
| *τ_decay_ (ms)* | | 7.1 ± 4.0 (26) | | 6.9 ± 3.3 (10) | | 0.97 (n.s.) | |
| sIPSC | |  | |  | |  | |
| *Frequency (Hz)* | | 1.6 ± 1.4 (26) | | 1.1 ± 0.7 (10) | | 0.28 (n.s.) | |
| *Amplitude (pA)* | | 25.8 ± 11.6 (26) | | 21.8 ± 7.3 (10) | | 0.32 (n.s.) | |
| *Rise_10-90%_ (ms)* | | 1.4 ± 0.7 (26) | | 1.7 ± 0.7 (10) | | 0.29 (n.s.) | |
| *τ_decay_ (ms)* | | 19.6 ± 8.5 (26) | | 20.9 ± 7.3 (10) | | 0.67 (n.s.) | |
| Values reported are mean ± standard deviation (*n*). *p<0.05; n.s., not significant (two-tailed unpaired *t* test). | | | | | | | |
